# Agro-Morphological Characterization and Nutritional Profiling of Traditional Himalayan Crop Landraces for Their Promotion Toward Mainstream Agriculture

**DOI:** 10.3389/fpls.2022.898220

**Published:** 2022-06-22

**Authors:** Nikhil Malhotra, Paras Sharma, Hemant Sood, Rahul Chandora, Mamta Arya, Jai Chand Rana, Mohar Singh

**Affiliations:** ^1^Indian Council of Agricultural Research (ICAR)-National Bureau of Plant Genetic Resources Regional Station, Shimla, India; ^2^Indian Council of Medical Research (ICMR)-National Institute of Nutrition, Hyderabad, India; ^3^Department of Biotechnology and Bioinformatics, Jaypee University of Information Technology, Waknaghat, India; ^4^Indian Council of Agricultural Research (ICAR)-National Bureau of Plant Genetic Resources Regional Station, Bhowali, India; ^5^Alliance of Bioversity International and CIAT, New Delhi, India

**Keywords:** landraces, agro-morphology, proximate composition, nutritional security, climate change

## Abstract

The northwest Indian Himalayas are often regarded as a *biological hotspot* for the presence of rich agro-biodiversity harboring locally adapted traditional crop landraces facing utter neglect owing to modern agricultural systems promoting high-yielding varieties. Addressing this challenge requires extricating the potential of such cultivars in terms of agro-morphological and nutritional attributes. In this study, 29 traditional crop landraces of maize (11), paddy (07), finger millet (03), buckwheat (05), and naked barley (03) were characterized and evaluated for target traits of interest. In maize, *Chitkanu* emerged as an early maturing landrace (107 days) with high concentrations of zinc (Zn), iron (Fe), and potassium (K), and *Safed makki* showed the highest 100-seed weight (28.20 g). Similarly, *Bamkua dhan* exhibited high concentrations of K and phosphorus (P), and *Lamgudi dhan* showed a high protein content (14.86 g/100 g) among paddy landraces. *Ogla-I* and *Phapra-I* showed high contents of protein (14.80 g/100 g) and flavonoids (20.50 mg/g) among buckwheat landraces, respectively, followed by *Nei-I*, which exhibited the highest protein content (15.66 g/100 g) among naked barley landraces. Most of the target traits varied significantly (*p* < 0.05) among evaluated samples, except those associated with finger millet landraces. The grouping pattern obtained by principal component analysis (PCA) and multidimensional scaling (MDS) was congruent with the geographical relationship among the crop landraces. This study led to the identification of elite crop landraces having useful variations that could be exploited in plant breeding programs and biofortification strategies for future crop improvement. Our endeavor would aid in conserving the depleting Himalayan agro-biodiversity and promoting versatile traditional crops toward mainstream agriculture vis-à-vis future nutritional security.

## Introduction

The broad spectrum of natural resources, including crop genetic resources, is under the immense burden of extinction mainly in the regions where cropping patterns are shifting at an alarming rate (Aguirre-Gutiérrez et al., 2017). The crop species inhabiting the northwest Himalayan region of India are scarcely distributed and have narrow scope to stride upward with changing climate due to the small area under cultivation (Kumari et al., 2017). This bottleneck has been created due to the loss of agro-biodiversity as a result of chemical-intensified agricultural farming and increasing replacement of locally adapted and traditionally grown cultivars by high-yielding modern varieties. With the human population estimated to cross ∼9 billion by the year 2050, there is an urgent requirement for an increase in food production. The traditional crops for food and agriculture are crucial in the fight against hunger and poverty and critical for the accomplishment of the Millennium Development Goals fixed for this purpose (Sachs et al., 2009; FAO, 2017). Besides this, they also play an important role in the long-term sustainability of any given agro-ecosystem by providing a plethora of opportunities for every research associated with genetic enhancement of crop varieties, namely, yield, quality, nutrition, and resistance to major biotic and abiotic stresses (Ebert, 2014; Tontisirin, 2014; Ba et al., 2018).

The substantial diversity in crop landraces makes them a good source of genes and alleles for plant breeding (Dwivedi et al., 2016). Therefore, it is pertinent to collect, conserve, and protect the traditional crop diversity, which is being maintained by the local farming communities since ancient times for their subsistence agriculture. An appropriate action at this stage would help to save these valuable genetic resources and provide opportunities to meet the future challenges of the growing demand for nutritional security. Under the changed regime of intellectual property rights, it is very important to protect our traditional crop landraces, farmer’s varieties, and associated agricultural knowledge thereof, so that disputes arising in terms of benefit sharing could be resolved and issues related to infringements and biopiracy may well be handled meticulously (Shiva et al., 2005; Barrueto et al., 2018). It is, therefore, imperative to characterize and evaluate traditional crops against target traits so that these could be promoted for mainstreaming into the agricultural production system. This will enhance the value of potential resources to not only improve farmer’s livelihood but also help in the generation of genomic resources for crop improvement. Therefore, the current emphasis was laid on nurturing the significance of traditional crop landraces from northwest Indian Himalayas by assessing their agro-morphological performance, including physicochemical parameters for the long-term sustainability of the fragile ecosystem.

## Materials and Methods

### Collection of Traditional Crop Landraces

Seeds of 29 different traditional landraces belonging to 5 crop groups, namely, maize (11), paddy (07), finger millet (03), buckwheat (05), and naked barley (03) were collected from different geographical locations in northwest Himalayan states of Himachal Pradesh and Uttarakhand during the summers of 2017–2019. These accessions were selected on the basis of their historical significance and maintenance by the farmers in the given agro-ecosystem. Detailed passport information regarding the genetic material has been provided in Table 1.

**TABLE 1:** Summary of the passport information of traditional crop landraces collected from northwest Himalayas of India.

Crop name	Botanical name	Vernacular name	Biological status	Type of material	Source	Frequency	Sample type	Sampling method	Habitat
Maize	*Zea mays*	*Safed challi*	Landrace	Seed	Farmer’s field	Abundant	Random	Selective	Cultivated
		*Ganga challi*	Landrace	Seed	Farmer’s field	Abundant	Random	Selective	Cultivated
		*Meethi challi*	Landrace	Seed	Farmer’s field	Abundant	Population	Random	Cultivated
		*Chitkanu*	Landrace	Seed	Farmer’s field	Abundant	Population	Selective	Cultivated
		*Lal makki*	Landrace	Seed	Farmer’s field	Rare	Population	Selective	Cultivated
		*Safed makki*	Landrace	Seed	Farmer’s field	Abundant	Population	Selective	Cultivated
		*Hacchi*	Landrace	Seed	Farm store	Abundant	Population	Random	Cultivated
		*Talaw makki*	Landrace	Seed	Farmer’s field	Abundant	Population	Selective	Cultivated
		*Chitri makki*	Landrace	Seed	Farmer’s field	Abundant	Population	Selective	Cultivated
		*Peeli kukdi*	Landrace	Seed	Farmer’s field	Abundant	Population	Random	Cultivated
		*Sathu kukdi*	Landrace	Seed	Farmer’s field	Abundant	Population	Random	Cultivated
Paddy	*Oryza sativa*	*Kaonoi dhan*	Landrace	Seed	Farmer’s field	Abundant	Population	Selective	Cultivated
		*Bamkua dhan*	Landrace	Seed	Farmer’s field	Abundant	Population	Selective	Cultivated
		*Lamgudi dhan*	Landrace	Seed	Farmer’s field	Abundant	Population	Selective	Cultivated
		*Dudhiya dhan*	Landrace	Seed	Farm store	Abundant	Population	Random	Cultivated
		*Safed phulpatas*	Landrace	Seed	Farmer’s field	Abundant	Random	Selective	Cultivated
		*Lal dhan*	Landrace	Seed	Farmer’s field	Abundant	Population	Selective	Cultivated
		*Batesu dhan*	Landrace	Seed	Farmer’s field	Abundant	Population	Selective	Cultivated
Finger millet	*Eleusine coracana*	*Madua early*	Landrace	Seed	Farmer’s field	Abundant	Population	Selective	Cultivated
		*Madua medium*	Landrace	Seed	Farmer’s field	Abundant	Population	Selective	Cultivated
		*Madua late*	Landrace	Seed	Farmer’s field	Abundant	Population	Selective	Cultivated
Buckwheat	*Fagopyrum esculentum/F. tataricum*	*Ogla-I*	Landrace	Seed	Farmer’s field	Abundant	Population	Selective	Cultivated
		*Ogla-II*	Landrace	Seed	Farm store	Abundant	Population	Selective	Cultivated
		*Ogla-III*	Landrace	Seed	Farmer’s field	Abundant	Population	Selective	Cultivated
		*Phapra-I*	Landrace	Seed	Farmer’s field	Abundant	Population	Random	Cultivated
		*Phapra-II*	Landrace	Seed	Farmer’s field	Abundant	Population	Selective	Cultivated
Naked barley	*Hordeum vulgare*	*Nanga jau*	Landrace	Seed	Farmer’s field	Occasional	Population	Selective	Cultivated
		*Nei-I*	Landrace	Seed	Farmer’s field	Abundant	Population	Selective	Cultivated
		*Nei-II*	Landrace	Seed	Farmer’s field	Abundant	Population	Selective	Cultivated

Demographics					Latitude	Longitude	Altitude (m)	Remarks (distinct features)	
Village	Block	District		State					

Tichi	Kullu	Kullu		Himachal Pradesh	31.9125	77.1146	1,168	High concentrations of minerals	
Tichi	Kullu	Kullu		Himachal Pradesh	31.9125	77.1146	1,168		
Majhoga	Tissa	Chamba		Himachal Pradesh	32.5036	76.3531	2,148	Maximum tassel branching	
Sanwal	Tissa	Chamba		Himachal Pradesh	32.5132	76.0859	2,005	Early maturity, high concentrations of minerals	
Ghari	Tissa	Chamba		Himachal Pradesh	32.5331	76.0818	2,034		
Sanwal	Tissa	Chamba		Himachal Pradesh	32.5132	76.0859	2,005	Maximum 100-seed weight	
Sanwal	Tissa	Chamba		Himachal Pradesh	32.5132	76.0859	2,005		
Talaw	Gopalpur	Mandi		Himachal Pradesh	31.5579	76.7641	903		
Hail	Tissa	Chamba		Himachal Pradesh	32.5601	76.1439	2,497	High concentrations of minerals	
Groan	Tissa	Chamba		Himachal Pradesh	32.5324	76.0740	1,744		
Majhoga	Tissa	Chamba		Himachal Pradesh	32.5031	76.0350	2,074		
Suri	Tarikhet	Almora		Uttarakhand	29.3422	79.30505	1,344		
Suri	Tarikhet	Almora		Uttarakhand	29.3422	79.30505	1,344	High concentration of nutrients and minerals	
Suri	Tarikhet	Almora		Uttarakhand	29.3422	79.30505	1,344	High concentration of nutrients and minerals	
Suri	Tarikhet	Almora		Uttarakhand	29.3422	79.30505	1,344		
Baggi	Mandi	Mandi		Himachal Pradesh	31.5777	76.9701	1,185		
Suri	Tarikhet	Almora		Uttarakhand	29.3422	79.30505	1,344		
Suri	Tarikhet	Almora		Uttarakhand	29.3422	79.30505	1,344		
Suri	Tarikhet	Almora		Uttarakhand	29.3422	79.30505	1,344		
Suri	Tarikhet	Almora		Uttarakhand	29.3422	79.30505	1,344		
Suri	Tarikhet	Almora		Uttarakhand	29.3422	79.30505	1,344		
Kamru	Sangla	Kinnaur		Himachal Pradesh	31.4348	78.2583	2,942	High content of proteins	
Sangla	Sangla	Kinnaur		Himachal Pradesh	31.4246	78.2646	2,678		
Themgarang	Sangla	Kinnaur		Himachal Pradesh	31.4166	78.2982	2,965		
Batseri	Sangla	Kinnaur		Himachal Pradesh	31.4072	78.3037	3,005	High amount of flavonoids	
Themgarang	Sangla	Kinnaur		Himachal Pradesh	31.4166	78.2982	2,965	High amount of flavonoids	
Sangla	Sangla	Kinnaur		Himachal Pradesh	31.4246	78.2646	2,678		
Lossar	Kaza	Lahaul & Spiti		Himachal Pradesh	32.4403	77.7476	4,031	High content of proteins	
Hansa	Kaza	Lahaul & Spiti		Himachal Pradesh	32.4516	77.8607	3,999	High content of proteins	

*For buckwheat and naked barley landraces, the vernacular names have been modified due to their similarity as Ogla-I, Ogla-II, Ogla-III, Phapra-I, and Phapra-II for buckwheat and Nei-I and Nei-II for naked barley landraces, respectively.*

### Agro-Morphological Characterization

All crop landraces were evaluated at the Experimental Farm of Indian Council of Agricultural Research (ICAR)-National Bureau of Plant Genetic Resources (NBPGR) Regional Station, Shimla (28^°^ 35′ N, 70^°^ 18′ E, altitude 1,924 m amsl) during summer season of 2018–2019 and 2019–2020. This study was performed in a Randomized Complete Block Design (RCBD) for 5 crop groups with each entry of the corresponding group replicated thrice, and recommended agronomic practices were followed to raise the experimental crops. The observations were recorded against important agro-morphological characters, namely, days to tasseling (DT), days to silking (DS), ear length (EL), ear height (EH), plant height (PH), days to 80% maturity (DM), and 100-seed weight (SW) for maize; days to 50% flowering (DF), leaf length (LL), leaf width (LW), number of tillers (NT), panicle height (PAH), PH, DM, and 100-SW for paddy; DF, finger length (FL), finger width (FW), NT, number of grains per spikelet (NGPS), PH, and DM for finger millet; DF, LL, LW, cyme length (CL), petiole length (PEL), PH, DM, and 1,000-SW for buckwheat; and days to emergence (DE), days to 50% heading (DH), NGPS, PH, DM and 1,000-SW for naked barley landraces, as per minimal descriptors developed by ICAR-NBPGR for individual crops under study (Mahajan et al., 2000). The quantitative analysis was determined by calculating the mean ± SD from triplicates (repeated thrice).

### Physicochemical Evaluation

Seed extracts of the analogous group were evaluated for biochemical analysis pertaining to proximate composition, secondary metabolites, and minerals. The quantitative analysis was determined by calculating the mean ± SD from triplicates (repeated thrice).

#### Determination of Moisture

An amount of 1 g of each sample was weighed in triplicates into stainless steel boxes. These were placed in a hot air oven at 60°C overnight for drying. Samples were taken out the next day and placed in the desiccators. The records were taken of the sample boxes after cooling to room temperature. The moisture content was calculated using the below-mentioned formula:


$$\text{Moisture }\left(\%\right)=\frac{\mathrm{initial}\,\mathrm{wt}-\mathrm{final}\,\mathrm{wt}}{\mathrm{wt}\,\mathrm{of}\,\mathrm{sample}}\text{×}100$$

#### Determination of Ash

Each sample was weighed in triplicates into a pre-weighed crucible. The crucibles along with samples were placed in a muffle furnace at 550°C overnight until white ash was obtained. The samples were taken out the next day and placed in the desiccator. The records of the samples were taken after cooling to room temperature. The weight of the residue amount of total ash per 100 g of the sample was calculated as follows:


$$\text{Ash }\,\left(\text{g}/100\,\text{g}\right)=\,\frac{\mathrm{final}\,\mathrm{wt}-\mathrm{initial}\,\mathrm{wt}}{\mathrm{wt}\,\mathrm{of}\,\mathrm{sample}}\,\text{×}\,100$$

#### Estimation of Protein and Fat

Proteins of maize, paddy, and millet landraces were estimated using the Kjeldahl method (gravimetric and titration) according to the AOAC protocol (AOAC, 2005). For buckwheat and naked barley landraces, the method developed by Lowry et al. (1951) was used to determine the protein at the absorbance of 610 nm using a spectrophotometer. Fat was also calculated following the AOAC method (AOAC, 2005) among all landraces.

#### Estimation of Carbohydrate

The presence of carbohydrates in maize landraces was assessed following the AOAC method (AOAC, 2005).

#### Estimation of Amylose and Amylopectin

Amylose and amylopectin contents in paddy landraces were analyzed enzymatically using the Megazyme Kit (Megazyme, Wicklow, Ireland) following the procedure mentioned in the provided booklet.^1^

#### Estimation of Phytic Acid

In paddy landraces, the phytate content was determined using the enzymatic method using the Megazyme Kit (Megazyme, Wicklow, Ireland) following the procedure mentioned in the booklet^2^. The absorbance of the colored solution was recorded at 655 nm using a spectrophotometer.

#### Estimation of Starch

In buckwheat and naked barley landraces, starch activity was measured as per the method described by Bernfeld (1955), wherein the reducing group liberated from starch was calculated by the reduction of 3,5-dinitrosalicylic acid.

#### Quantification of Flavonoids

Flavonoids were quantified by reverse phase-high performance liquid chromatography (RP-HPLC) in buckwheat and naked barley landraces. The samples were analyzed with the Waters 515 HPLC system equipped with a model 515 solvent pump, an ASI-100 autosampler, a PDA-Waters 2,996 detector, Waters In-line degasser AF, and Empower Pro software. Flavonoids were detected at 350 nm by comparison of their retention times with those of pure standards and individual quantification. Quantification was performed using the linear calibration curves of standard compounds.

#### Quantification of Minerals

Minerals were quantified using the Inductively Coupled Plasma-Optical Emission Spectrometry (ICP-OES) iCAP™ 7200 ICP-OES Analyzer (Thermo Scientific). In brief, samples were digested by acid hydrolysis using nitric acid followed by microwave digestion. After appropriate dilution, minerals comprising trace and heavy metals, including zinc (Zn), copper (Cu), iron (Fe), manganese (Mn), magnesium (Mg), potassium (K), phosphorus (P), chromium (Cr), nickel (Ni), cobalt (Co), selenium (Se), and cadmium (Cd), were evaluated in all landraces.

### Statistical Analysis

The study data were assessed with a combination of descriptive techniques and were tested for significant levels at *p* < 0.05 using the multivariate multiple regression. Furthermore, it was analyzed by principal component analysis (PCA) and multidimensional scaling (MDS) using XLSTAT version 2016.02 (Addinsoft SARL, France).

## Results

The quantitative data were determined using range, mean, standard error, and coefficient of variation for different agro-morphological characters and nutritional profiles assessed among traditional crop landraces (Table 2).

**TABLE 2:** Comparative performance of different agro-morphological and nutritional traits of traditional crop landraces.

Crop name	Descriptor	Min	Max	Mean	Standard error	Coefficient of variation (%)
Maize	Days to tasseling	55	71	65.27	1.54	7.84
	Days to silking	58	81	71.91	2.08	9.6
	Ear length (cm)	18.34	29.89	25.91	0.96	12.28
	Ear height (cm)	63.41	149.76	115.54	8.74	25.09
	Plant height (cm)	158.26	275.5	215.52	11.17	17.19
	Days to 80% maturity	107	127	118.64	1.9	5.32
	100-seed weight (g)	17.41	28.2	23.35	0.83	11.76
	Ash (g/100 g)	1.21	5.95	2.75	0.46	55.32
	Fat (g/100 g)	3.93	7.79	6.63	0.31	15.66
	Moisture (%)	4.03	5.98	5.11	0.18	11.63
	Protein (g/100 g)	9.46	12.62	10.47	0.31	9.68
	Carbohydrate (g/100 g)	49.02	63.91	59.39	1.23	6.89
Paddy	Days to 50% flowering	44	102	91.43	7.99	23.13
	Leaf length (cm)	35.8	57.5	50.48	2.92	15.33
	Leaf width (cm)	1.3	1.75	1.58	0.06	9.6
	No. of tillers	7	13	11	0.76	18.18
	Panicle height (cm)	21.5	24.79	22.87	0.59	6.8
	Plant height (cm)	75.1	157.68	132.32	10.4	20.8
	Days to 80% maturity	73	153	138.43	10.99	21
	100-seed weight (g)	1.62	2.85	2.41	0.16	17.78
	Ash (g/100 g)	0.85	1.22	1.01	0.05	14.26
	Fat (g/100 g)	2.52	3.56	2.88	0.13	12.19
	Moisture (%)	4.27	7.14	6.22	0.36	15.41
	Protein (g/100 g)	9.27	14.86	12.26	0.7	15.21
	Amylose (%)	16.45	36.74	27.88	3.38	32.08
	Amylopectin (%)	63.26	83.54	72.11	3.38	12.4
	Phytic acid (g/100 g)	0.67	1.2	0.96	0.08	21.77
Finger millet	Days to 50% flowering	89	100	94.33	3.18	5.84
	Finger length (mm)	411.68	423.14	416.69	3.39	1.41
	Finger width (mm)	19.18	21.87	20.86	0.84	7.01
	No. of tillers	5	5	5	0	0
	No. of grains per spikelet	145	152	149	2.08	2.42
	Plant height (cm)	115.78	121.63	118.68	1.69	2.46
	Days to 80% maturity	119	128	124	2.65	3.7
	Ash (g/100 g)	2.66	3.2	2.9	0.16	9.43
	Fat (g/100 g)	2.79	3.41	3.08	0.18	10.16
	Moisture (%)	5.06	5.52	5.28	0.14	4.44
	Protein (g/100 g)	8.41	8.66	8.54	0.07	1.47
Buckwheat	Days to 50% flowering	43	50	46.2	1.28	6.2
	Leaf length (cm)	7.11	10.45	8.65	0.61	15.84
	Leaf width (cm)	8.31	12.05	10.05	0.79	17.58
	Cyme length (cm)	2.75	4.33	3.16	0.3	20.99
	Petiole length (cm)	6.98	9.11	8.34	0.38	10.15
	Plant height (cm)	119.82	143.66	132.35	4.26	7.19
	Days to 80% maturity	90	112	102.4	3.64	7.95
	1,000-seed weight (g)	17.49	22.64	20.24	0.85	9.44
	Ash (g/100 g)	1.7	2	1.86	0.05	6.13
	Fat (g/100 g)	5.1	7	5.88	0.42	16.02
	Moisture (%)	7.9	9.9	9	0.4	9.88
	Protein (g/100 g)	10.11	14.8	12.02	0.88	16.36
	Starch (U/μM/30 min)	4.77	6.12	5.32	0.3	12.55
	Flavonoids (mg/g)	2.99	20.5	9.51	3.94	92.7
Naked barley	Days to emergence	8	13	10	1.53	26.46
	Days to 50% heading	95	108	99.33	4.33	7.56
	No. of grains per spikelet	62	71	67	2.65	6.84
	Plant height (cm)	95.22	100.37	98.57	1.68	2.95
	Days to 80% maturity	140	150	143.67	3.18	3.83
	1,000-seed weight (g)	38.15	45.02	42.38	2.14	8.74
	Ash (g/100 g)	1.85	2.9	2.23	0.34	26.23
	Fat (g/100 g)	2.51	3.86	2.96	0.45	26.27
	Moisture (%)	2.88	5.82	3.9	0.96	42.54
	Protein (g/100 g)	12.35	15.66	14.47	1.06	12.73
	Starch (U/μM/30 min)	13.5	15.2	14.57	0.54	6.38
	Flavonoids (mg/g)	0.2	0.5	0.4	0.1	43.3

### Agro-Morphological Characterization

**FIGURE 1:**
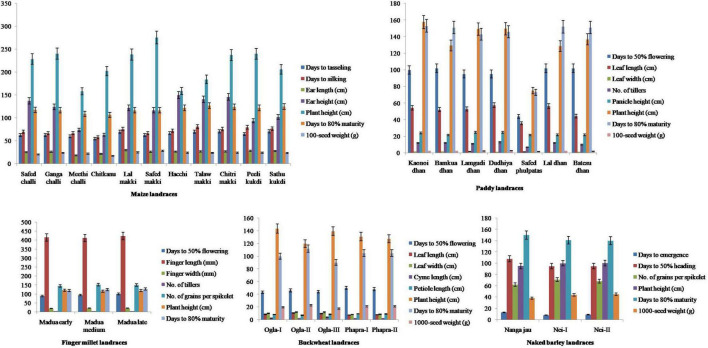
Agro-morphological characterization of traditional crop landraces from northwest Indian Himalayas. *Error bars* represent mean ± SD for data recorded in triplicates (repeated thrice).

All the traditional crop landraces were characterized by important agro-morphological traits as described in Figure 1. Results revealed that maize landraces differed significantly (*p* < 0.05) in terms of EL (18.34–29.89 cm), EH (63.41–149.76 cm), PH (158.26–275.50 cm), and 100-SW (17.41–28.20 g). A high coefficient of variation was recorded for EH (25.09%) followed by EL (12.28%), PH (17.19%), and 100-SW (11.76%), which showed moderate variability. Inexplicably, DM showed low variability (5.32%) where *Chitkanu* (107 days) and *Talaw makki* (127 days) emerged as early and late maturing landraces, respectively. The maximum 100-SW was found in *Safed makki* (28.20 g). In contrast, paddy landraces revealed significant variations (*p* < 0.05) for DF (44-102), LL (35.80–57.50 cm), NT (7-13), PH (75.10–157.68 cm), DM (73–153), and 100-SW (1.62–2.85 g) with moderate variations for DF (23.13%), LL (15.33%), NT (18.18%), PH (20.80%), DM (21.00%), and 100-seed (17.78%). *Safed phulpatas* exhibited early maturity (73 days) while *Kaonoi dhan* matured late (153 days) as compared with others. Similarly, buckwheat landraces showed a comparable pattern for LL (7.11–10.45 cm), LW (8.31–12.05 cm), and CL (2.75–4.33 cm) with moderate variability of 15.84, 17.58, and 20.99% for respective traits. Interestingly, these also exhibited low variation (7.95%) for DM. The early and late maturity was observed in *Ogla-III* (90 days) and *Ogla-II* (112 days), respectively. Notably, less significant variations (*p* < 0.05) for the aforementioned characters were observed in finger millet and naked barley landraces, except for DE in the latter, where a high level of variation (26.46%) was recorded. Furthermore, the data were projected into first and second principal components (PC1 and PC2) to illustrate intra- and inter-landrace variations. It was observed that PC1 interacted with PC2 in a similar pattern among all crop landraces, and the contribution of PC1 was more than PC2. The total variability of 100% indicated successful variance of uncorrelated components without loss of any information. PCA suggested that different traits contributed significantly to the total variation assessed in all crop landraces except for finger millet, namely, DT, DS, EL, EH, and DM in maize; DF, LL, LW, NT, PH, DM, and 100-SW in paddy; DF and PEL in buckwheat; and DE, DE, and DM in naked barley landraces (Supplementary Figure 1).

### Proximate Analysis

**FIGURE 2:**
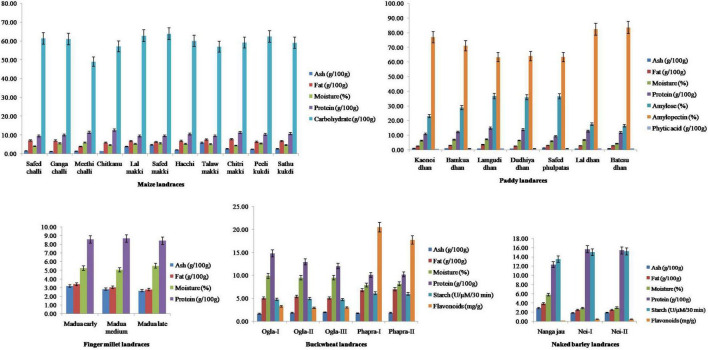
Comparison of nutritional attributes of traditional crop landraces from northwest Indian Himalayas. *Error bars* represent mean ± SD for data recorded in triplicates (repeated thrice).

All seed extracts exhibited affirmative results in the presence of various biochemicals (Figure 2). Ash content varied significantly (*p* < 0.05) among the evaluated samples of maize landraces. The highest and lowest ash contents were observed in *Talaw makki* (5.95 g/100 g) and *Ganga challi* (1.21 g/100 g), respectively, with very high variability (55.32%). Fat content ranged from 3.93 g/100 g in *Meethi challi* to 7.79 g/100 g in *Chitri makki* with moderate variation (15.66%). Among paddy landraces, moderate variability was recorded for ash (14.26%), fat (12.19%), and moisture (15.41%) contents. Protein content differed significantly (*p* < 0.05) and ranged from 9.27 g/100 g in *Safed phulpatas* to as high as 14.86 g/100 g in *Lamgudi dhan* with moderate variability (15.21%). Amylose content varied significantly (*p* < 0.05) and was observed between 16.45% in *Batesu dhan* to 36.74% in *Lamgudi dhan* (32.08%). Similarly, phytic acid content showed a high variation (21.77%) and significantly differed (*p* < 0.05) from 0.67 g/100 g in *Kaonoi dhan* to 1.20 g/100 g in *Batesu dhan*. Furthermore, among buckwheat landraces, fat (16.02%) and starch (12.55%) contents showed moderate variability. Protein content ranged from 10.11 g/100 g in *Phapra-I* to 14.80 g/100 g in *Ogla-I* with moderate variation (16.36%). Interestingly, a wide range of significant variation (*p* < 0.05) was observed for the content of flavonoids, which extended from 2.99 mg/g in *Ogla-II* to the highest 20.50 mg/g in *Phapra-I* with maximum variation (92.70%). Among naked barley landraces, protein content varied significantly (*p* < 0.05) and ranged from 12.35 g/100 g in *Nanga jau* to 15.66 g/100 g in *Nei-I* with moderate variation (12.73%). Moreover, these also exhibited high variability percentages for ash, fat, moisture, and flavonoid contents. In contrast, significant variations (*p* < 0.05) for nutritional traits were found to be least among finger millet landraces. Interestingly, PCA indicated substantial variance among contributing traits for all crop landraces, namely, ash and fat in maize; protein in paddy; ash and fat in finger millet; fat, starch, and flavonoids in buckwheat; and ash, fat, and moisture in naked barley landraces (Supplementary Figure 2).

### Mineral Composition

A wide range of variability was observed for different minerals in all crop landraces (Table 3). However, we focused on trace elements, such as Zn, Fe, K, and P, owing to their role in future nutritional security. Among maize landraces, Zn content varied significantly (*p* < 0.05) where *Chitkanu* (3.13 mg/100 g) and *Peeli kukdi* (1.88 mg/100 g) showed the highest and the lowest Zn contents, respectively. Similarly, Fe content varied widely among the evaluated samples and ranged from 0.61 mg/100 g in *Hacchi* to as high as 2.74 mg/100 g in *Chitkanu*. K content varied significantly (*p* < 0.05) among the maize landraces and ranged between 299.96 and 529.02 mg/100 g. The lowest and the highest K contents were observed in *Hacchi* and *Chitkanu*, respectively. Moreover, P content varied significantly (*p* < 0.05) with the highest and the lowest found in *Peeli kukdi* (407.61 mg/100 g) and *Hacchi* (135.40 mg/100 g), respectively. Furthermore, significant differences (*p* < 0.05) for K and P contents were observed in paddy landraces as compared with Zn and Fe contents. *Bamkua dhan* exhibited the highest K (467.77 mg/100 g) and P (249.96 mg/100 g) contents along with *Batesu dhan* (310.65 mg/100 g) and *Lal dhan* (112.50 mg/100 g) which had the lowest K and P contents, respectively. On the contrary, fewer variations were observed for selected trace elements among finger millet, buckwheat, and naked barley landraces, although these showed adequate concentrations of target minerals.

**TABLE 3:** Comparison of mineral composition of traditional crop landraces.

Crop name	Landrace	Mineral composition^#^											
		Zn	Cu	Fe	Mn	Mg	K	P	Cr	Ni	Co	Se	Cd
Maize	*Safed challi*	2.55	0.22	1.00	0.42	138.39	440.78	377.80	73.71	86.57	1.05	0.10	0.29
	*Ganga challi*	2.16	0.27	1.93	0.37	133.12	309.49	354.72	32.77	6.02	0.38	0.09	0.20
	*Meethi challi*	2.07	0.51	1.31	0.44	160.11	435.28	317.05	35.47	3.26	0.34	0.07	0.14
	*Chitkanu*	3.13	0.35	2.74	0.62	174.50	529.02	193.16	37.14	56.63	1.24	0.49	7.04
	*Lal makki*	2.66	0.32	1.49	0.65	125.87	387.39	219.68	34.58	117.24	2.16	0.93	3.51
	*Safed makki*	2.05	0.26	1.28	0.47	138.29	476.06	300.49	46.89	28.00	0.77	0.36	0.42
	*Hacchi*	1.93	0.42	0.61	0.35	105.68	299.96	135.40	57.00	54.97	3.31	0.47	0.22
	*Talaw makki*	2.09	0.39	1.36	0.49	99.27	355.59	215.05	40.83	7.87	0.63	0.50	0.72
	*Chitri makki*	2.36	0.30	1.14	0.43	167.43	404.73	268.52	40.17	8.43	0.54	0.16	0.21
	*Peeli kukdi*	1.88	0.21	0.79	0.40	161.07	436.98	407.61	31.40	48.36	15.77	0.24	0.71
	*Sathu kukdi*	2.15	0.26	1.20	0.37	115.92	392.05	288.42	110.73	84.94	1.11	0.21	0.07
Paddy	*Kaonoi dhan*	2.41	0.95	0.95	0.95	115.83	452.87	176.53	−	−	−	−	−
	*Bamkua dhan*	2.33	0.82	0.82	0.82	132.73	467.77	249.96	−	−	−	−	−
	*Lamgudi dhan*	2.35	0.82	0.82	0.82	113.12	399.21	211.54	−	−	−	−	−
	*Dudhiya dhan*	2.35	1.45	1.45	1.45	89.95	325.15	143.28	−	−	−	−	−
	*Safed phulpatas*	2.19	1.59	1.59	1.59	104.08	350.56	176.61	−	−	−	−	−
	*Lal dhan*	2.06	1.01	1.01	1.01	90.16	311.92	112.50	−	−	−	−	−
	*Batesu dhan*	2.10	0.94	0.94	0.94	88.20	310.65	113.64	−	−	−	−	−
Finger millet	*Madua early*	2.29	1.87	3.04	10.49	177.15	318.85	284.88	27.47	7.05	0.36	0.12	0.24
	*Madua medium*	1.99	1.13	2.01	13.53	216.21	558.40	246.81	74.70	33.31	4.92	0.13	0.17
	*Madua late*	1.85	1.03	2.55	13.17	182.77	716.51	207.62	28.12	84.08	2.17	0.48	1.09
Buckwheat	*Ogla-I*	2.31	1.25	2.07	0.43	225.86	447.87	389.95	−	−	−	−	−
	*Ogla-II*	2.73	1.25	2.07	0.52	208.50	481.34	354.15	−	−	−	−	−
	*Ogla-III*	2.33	1.25	2.07	0.44	210.38	435.69	385.74	−	−	−	−	−
	*Phapra-I*	2.65	1.41	1.99	0.12	276.41	510.25	402.55	−	−	−	−	−
	*Phapra-II*	2.65	1.41	2.00	0.20	270.59	555.48	411.23	−	−	−	−	−
Naked barley	*Nanga jau*	2.10	1.69	27.70	1.28	117.89	581.68	222.09	33.33	9.62	0.35	0.13	0.19
	*Nei-I*	2.42	1.81	30.05	1.18	115.32	603.52	257.66	30.85	7.45	0.40	0.08	0.28
	*Nei-II*	2.41	1.75	29.99	1.21	115.13	600.45	251.08	31.12	8.03	0.33	0.11	0.24

*^#^Data recorded in triplicates (repeated thrice) and represented as mean ± SD, conc. in mg/100 g.*

## Discussion

The ever-increasing reliance on major crops has typical agronomic, ecological, nutritional, and economic risks and is possibly unsustainable in the long run, especially in view of nutritional security. Traditional crop landraces constitute the base of diversity in indigenous communities of a developing country and offer greater system resilience as per futuristic needs. The variability of quantitative traits in any crop is highly influenced by genetic and environmental factors as well as interactions among them, whereas uniformity of individuals and stability of quantitative traits are major requirements for the development of improved cultigens (Xu et al., 2017). In this study, traditional landraces of target crops were characterized and evaluated for their usefulness in mainstream agriculture and maintenance of depleting genetic diversity in the Himalayas. Among maize landraces, the most variable agro-morphological traits were EL, EH, PH, and 100-SW. Several studies across the globe have highlighted the genetic variability in maize germplasm (Prasanna, 2012). Kumar et al. (2015) also reported greater variability for many of these traits in maize landraces collected from the northwest Himalayan region. Likewise, in paddy landraces, the most variable agro-morphological traits were DF, LL, NT, PH, DM, and 100-SW. Manjunatha et al. (2018) and Manohara et al. (2018) had also shown similar phenotypic diversity among rice landraces. Moreover, these findings were also in concordance with a recent study on Turkish rice landraces (Konak et al., 2021). Furthermore, buckwheat landraces showed a related pattern of morphological variations for LL, LW, and CL. Previous studies by Facho et al. (2016) and Misra et al. (2019) have also reported morphological diversity among indigenous buckwheat germplasm. However, naked barley landraces exhibited a high degree of variability for DE only, even though earlier studies by Yadav et al. (2018) and Karkee et al. (2020) have reported greater agro-morphological variations among these landraces. Such a wide range of variability among traditional crop landraces was caused by the fact that phenotypic expression is governed by complex traits and developmental stage-specific systems (Cobb et al., 2019). Moreover, the open-pollinated nature of landraces with specific adaptation to local conditions and the continuous use of seeds maintained by the farmers could result in high agro-morphological variability among them.

Moreover, all crop landraces showed comparable variability for the majority of nutritional traits. Among maize landraces, ash and fat were found to be the greatest variable traits. On the contrary, Kumar et al. (2015) reported significant differences among maize landraces from the northwest Himalayas for fat and sugar contents. These variances are mainly associated with a difference in their geographical origin and genetic diversity among selected maize genotypes (Kumar et al., 2015). We identified elite maize landraces as having higher concentrations of carbohydrate, fat, and protein contents, making our findings readily comparable with previous reports by Suri and Tanumihardjo (2016) and Saeed et al. (2021) on the nutraceutical properties of the maize. Amylose and phytic acid emerged as the most variable traits among paddy landraces followed by moisture, protein, ash, and fat. Phytic acid is the major antinutrient present in rice that is responsible for chelating the divalent ions (Fe and Zn) that reduce their bioavailability (Perera et al., 2018). A recent review by Marolt and Kolar (2021) suggested that the methods of analysis and genotype significantly affect the phytic acid content in paddy. These results were also comparable with nutritional diversity among rice collections from Kashmir Himalayas (Ashraf et al., 2017). In contrast, a greater diversity for most nutrients was observed among buckwheat and naked barley landraces. Both these crops are known to contain various phytochemicals in their seeds namely protein, starch, lipid, and secondary metabolites (Asare et al., 2011; Singh et al., 2020). The contents of these compounds are largely influenced by different factors, such as species and environmental conditions (Lee et al., 2016). Our results revealed that buckwheat landraces had variability in their biochemical profiles, especially for the contents of protein and flavonoids. The data obtained were in agreement with earlier findings by Przybylski and Gruczynska (2009) and Tang et al. (2011) that were also reviewed by Singh et al. (2020) recently. The identification of buckwheat landraces for high contents of protein and flavonoids could be a major finding for discerning sources of useful traits among buckwheat germplasm globally. Similar results were also obtained in naked barley landraces. The existing diversity in the biochemical composition of crop landraces may be attributed to the disparity in the genetic makeup of cultivars and growing conditions.

Likewise, the ever-increasing problem of micronutrient malnutrition, commonly known as *hidden hunger*, has been affecting lives across the world. The conventional strategies to improve future nutritional security include dietary diversification (Jha and Warkentin, 2020) and biofortification of crops (Bouis and Saltzman, 2017; Garg et al., 2018). Our study also revealed the presence of vital components, such as Zn, Fe, K, and P, among maize and paddy landraces. A wide range of variability was observed for the aforementioned minerals among them. Our results were found to be in conjunction with previous reports by Suri and Tanumihardjo (2016) and Saeed et al. (2021) on the biochemical properties of maize along with studies by Deb et al. (2015) and Ray et al. (2021) on traditional rice landraces of India. Maganti et al. (2020) also reported considerable variation in Fe and Zn contents in traditional rice genotypes. Several studies have also shown substantial variations in the concentration of minerals in other crops (White and Broadley, 2009; Garg et al., 2018). Reports have suggested that biofortification through plant breeding could be effectively utilized as a sustainable approach to improve the nutritional profile of food crops by utilizing cues from versatile crop genotypes (Carvalho and Vasconcelos, 2013; Pratap et al., 2021).

**FIGURE 3:**
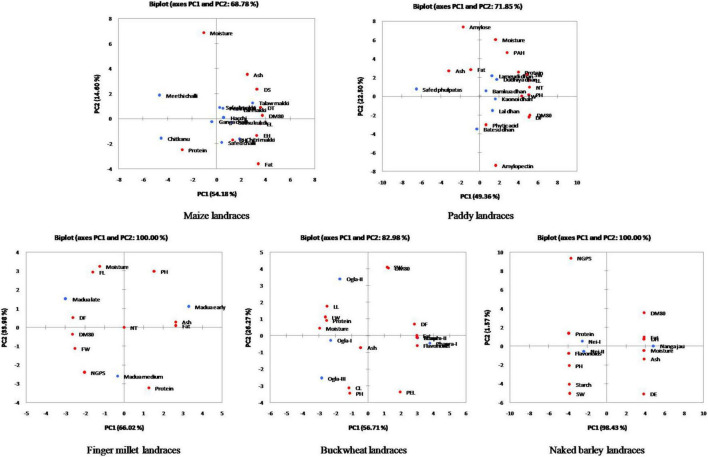
Principal component analysis (PCA) plots of agro-morphological characteristics and nutritional attributes for traditional crop landraces along with the projection of bi-plot scores.

**FIGURE 4:**
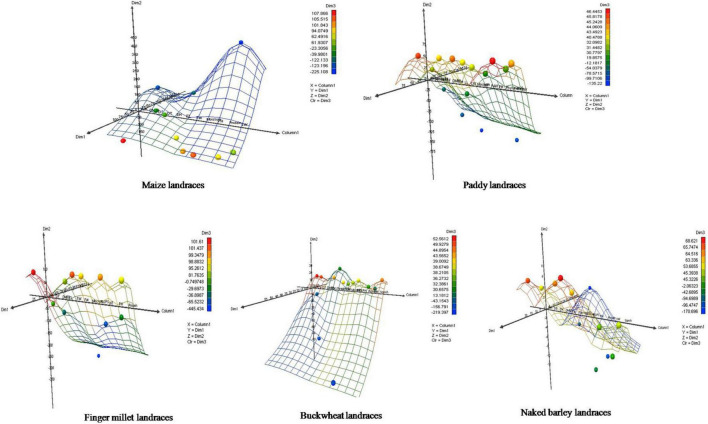
Multidimensional scaling of agro-morphological characteristics and nutritional attributes for traditional crop landraces. *X* represents column 1, *Y* represents dimension 1, *Z* represents dimension 2, and *Color* represents dimension 3.

Furthermore, the relative contribution of each trait among crop landraces was inferred by PCA and MDS. PCA was used to visualize the association between variables and determined the non-correlated factors that are linear combinations of initial variables. Bi-plots were generated for PCA analysis of all the observations as described in Figure 3. Among all crop landraces, maximum variability of 54.18, 49.36, 66.02, 56.71, and 98.43% was exhibited by PC1 in maize, paddy, finger millet, buckwheat, and naked barley landraces, respectively, with the corresponding contributing traits that showed high variations. Similarly, 14.60, 22.50, 33.98, 26.27, and 1.57% variabilities were explained by PC2 in the aforementioned landraces, respectively, with the corresponding contributing traits, which exhibited diversity. To consolidate these findings, MDS was performed to measure the disparities and residual distances among various traits in all crop landraces. Euclidean distances were calculated using quantitative data, and 3-dimensional plots were generated for analyzing the relationship among combined data sets (Figure 4). The comparative analysis revealed the association of many agro-morphological and nutritional traits with each other, which may influence region-specific differences among all crop landraces (Supplementary Table 1). Overall, this study led to the identification of elite crop landraces (Table 4) carrying desired traits of interest that could be introgressed into the background of existing cultivars for their genetic improvement. This would, in turn, may also help in promoting them toward mainstream agriculture.

**TABLE 4:** Identification of elite traditional crop landraces for mainstream agriculture.

Crop	Landrace	Area	Target trait(s)	
Maize	*Chitkanu*	Sanwal, Chamba, Himachal Pradesh	Early maturity (107 days), high concentrations of Zn, Fe, and K	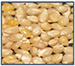
	*Safed makki*	Sanwal, Chamba, Himachal Pradesh	Maximum 100-seed weight (28.20 g)	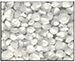
Paddy	*Bamkua dhan*	Suri, Almora, Uttarakhand	High concentrations of K and P	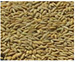
	*Lamgudi dhan*	Suri, Almora, Uttarakhand	High content of protein (14.86 g/100 g)	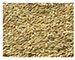
Buckwheat	*Ogla-I*	Kamru, Kinnaur, Himachal Pradesh	High content of protein (14.80 g/100 g)	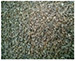
	*Phapra-I*	Batseri, Kinnaur, Himachal Pradesh	High amount of flavonoids (20.50 mg/g)	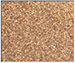
Naked barley	*Nei-I*	Lossar, Lahaul and Spiti, Himachal Pradesh	High content of protein (15.66 g/100 g)	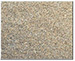

This study demonstrates a unique pattern of phenotypic diversity and nutritional features among crop landraces in the northwest Himalayas of India. The genetic relationships revealed by PCA as well as MDS were largely in agreement with their geographical distribution and provided a more comprehensive insight into the inherent association among target traits. These findings led to the identification of elite genotypes having desired traits of interest, which could be used in future crop improvement programs to develop new crop varieties. Besides, the landraces possessing high variation in concentrations of vital trace elements may be utilized to broaden the genetic base of existing cultigens. Finally, promoting the use of traditional crop landraces and revisiting the diverse areas harboring them would help in the conservation and long-term sustainability of otherwise forgotten traditional agriculture system, which will also aid in realizing futuristic goals of nutritional security.

## Data Availability Statement

The original contributions presented in this study are included in the article/Supplementary Material, further inquiries can be directed to the corresponding author.

## Author Contributions

MS conceptualized the study, contributed to writing, reviewing, and editing the manuscript, and performed funding acquisition. NM contributed to investigation, formal analysis, and writing the original draft. PS and HS contributed to methodology, data curation, and resources. RC and MA contributed to methodology and resources. JR contributed to writing, reviewing, and editing the manuscript. All authors contributed to the article and approved the submitted version.

## Conflict of Interest

The authors declare that the research was conducted in the absence of any commercial or financial relationships that could be construed as a potential conflict of interest.

## Publisher’s Note

All claims expressed in this article are solely those of the authors and do not necessarily represent those of their affiliated organizations, or those of the publisher, the editors and the reviewers. Any product that may be evaluated in this article, or claim that may be made by its manufacturer, is not guaranteed or endorsed by the publisher.

## Footnotes

1
https://www.megazyme.com/documents/Assay_Protocol/K-AMYL_DATA.pdf

2
https://www.megazyme.com/documents/Assay_Protocol/K-PHYT_DATA.pdf
