# Supplementary material for: Agro-Morphological Characterization and Nutritional Profiling of Traditional Himalayan Crop Landraces for Their Promotion Toward Mainstream Agriculture
Source: Front Plant Sci. 2022 Jun 22;13:898220. doi: 10.3389/fpls.2022.898220 (PMC9258745; doi:10.3389/fpls.2022.898220)
Supplement: Supplementary file 1 [file Data_Sheet_1.docx]

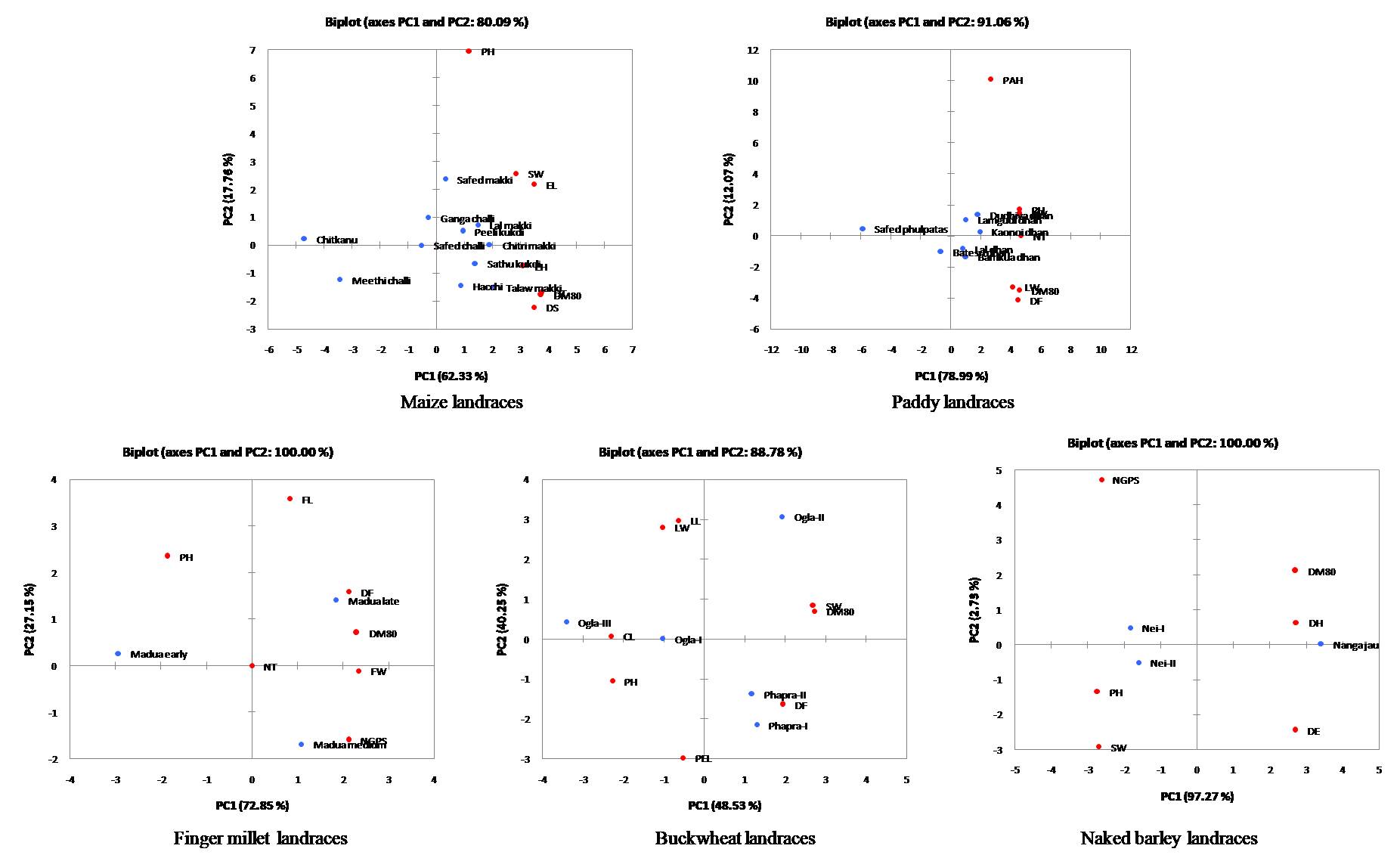


**SUPPLEMENTARY FIGURE S1** PCA analysis showing variability in agro-morphological traits among traditional crop landraces of northwest Indian Himalayas.


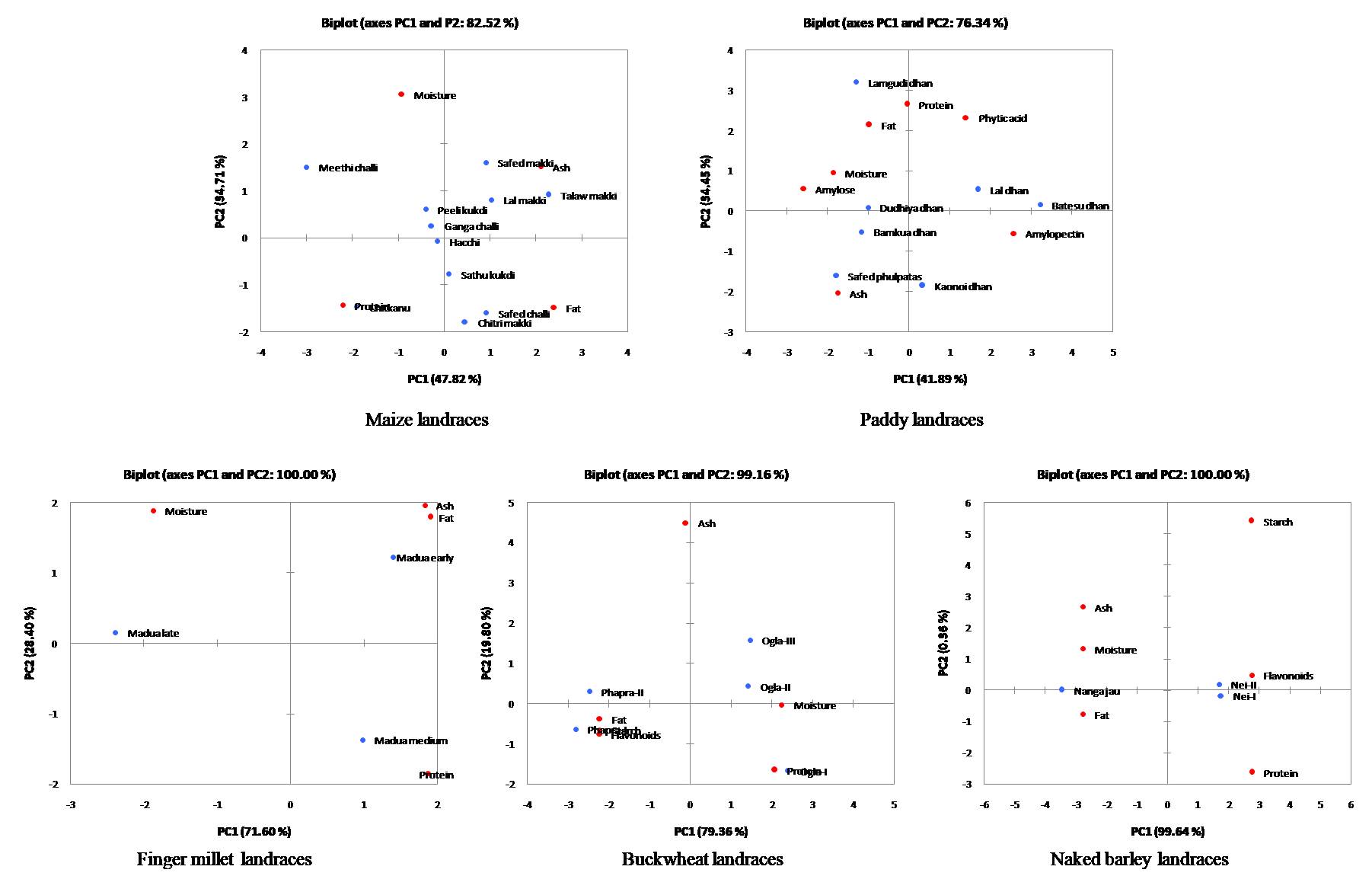


**SUPPLEMENTARY FIGURE S2** PCA analysis showing variability in nutritional traits among traditional crop landraces of northwest Indian Himalayas.
